# Diagnosis of posterior staphyloma using the radius of steepest curvature among retinal pigment epithelium segmentation line measured by optic coherent tomography

**DOI:** 10.1186/s12886-024-03321-z

**Published:** 2024-02-07

**Authors:** Sunho Park, Keunheung Park, Sangcheol Yang, Ik Soo Byon, Ji Eun Lee, Sung Who Park

**Affiliations:** 1 Department of Ophthalmology, School of Medicine, Pusan National University and Medical Research Institute, Pusan National University Hospital, 1-10 Ami-dong, Seo-gu, 602-739 Pusan, South Korea; 2 Crystal Eye Clinic , Pusan, Republic Of Korea

**Keywords:** Diagnosis, Curvature, OCT, Posterior staphyloma, Pathologic myopia, Radius, Retinal pigment, Epithelium

## Abstract

**Purpose:**

To investigate a novel marker to diagnose posterior staphylomas by measuring the radius of the steepest curvature on the retinal pigment epithelium (RPE) segmentation line using optical coherence tomography (OCT).

**Study Design:**

Retrospective Cross-sectional Study.

**Methods:**

The authors developed a prototype software to measure the radius of curvature on the RPE segmentation line of OCT. Twelve images of 9-mm radial OCT scans were used. The radius of curvature was measured at the steepest area of the RPE segmentation line, and the macular curvature (MC) index was calculated based on its reciprocal. Based on the wide-field fundus findings, the study sample was divided into three groups: definite posterior staphyloma, no posterior staphyloma, and undetermined. The differences of MC index among the groups and the correlation between the MC index, age, and axial length were analyzed.

**Results:**

The present study analyzed 268 eyes, with 54 (20.1%) with definite posterior staphyloma, 202 (75.4%) with no posterior staphyloma, and 12 (4.5%) with undetermined disease status. A maximum MC index of 37.5 was observed in the group with no posterior staphyloma, which was less than the minimum MC index of 42.7 observed in the group with definite posterior staphyloma. The MC index had strong correlations with the axial length and age in eyes with high myopia.

**Conclusions:**

Eyes with posterior staphyloma have a steeper curvature than those with radius 8.44 mm, while eyes without posterior staphyloma do not. MC index 40 (radius 8.44 mm) might act as a reference to distinguish between those with and those without posterior staphyloma.

**Supplementary Information:**

The online version contains supplementary material available at 10.1186/s12886-024-03321-z.

## Introduction

The prevalence of myopia is rapidly increasing worldwide [1, 2]. Myopic eyes with very long axial lengths or a high degree of myopic refractive error, like 26 mm or longer or -6 diopter or less are classified as eyes with high myopia [3–6]. Pathologic myopia is defined as myopia having pathologic changes related to myopia, and posterior staphyloma is a key component of these pathologic changes [7–9]. However, no consensus has yet been established regarding the diagnosis of posterior staphyloma.

Posterior staphyloma is a structural deformity of the eyeball defined as “an outpouching of the posterior wall of the eye” [10]. Fundus photography [4, 11–14], ultrasonography [3, 15], three-dimensional magnetic resonance imaging (3D MRI) [14], and ultra-widefield optical coherent tomography (OCT) [3, 16–18] have been used to detect posterior staphyloma through the identification of inflection points.

However, posterior staphyloma is typically a progressive condition [3, 16, 19]. As inflection lines are difficult to detect during the early stages of development, their utility in the early diagnosis of posterior staphyloma remains limited [16]. Additionally, the method of identifying inflection is not suitable for monitoring the progression or change in posterior staphyloma.

Posterior staphyloma, a deformity of the posterior eyeball, has a steep curvature of the retinal pigment epithelium (RPE) segmentation line in OCT. We hypothesized that the radius of curvature might be a marker that reflects the severity of posterior staphyloma. We measured the radius of the steepest curvature on the RPE segmentation line and attempted to utilize the radius to detect posterior staphyloma and to follow up its progression.

## Methods

The Institutional Review Board of Pusan National University Hospital approved the study protocol (1901-011-075), which complied with the tenets of the Declaration of Helsinki. The current retrospective study included 268 patients who underwent swept-source OCT (Atlantis or Triton, Topcon, Japan) imaging with 12 9-mm radial scans and IOL master (Carl Zeiss Meditec, Dublin, CA) to measure axial length between January 2015 and December 2018. Twelve 9 mm-radial OCT scan of patients whose eyes had information of axial lengths measured by IOL master were reviewed. Eyes with macular edema or a history of vitreoretinal surgery were excluded from the study. Epiretinal membrane that seems stable was not excluded. Among patients with two eligible eyes for inclusion, the eye with the longer axial length was included.

**Fig. 1:**
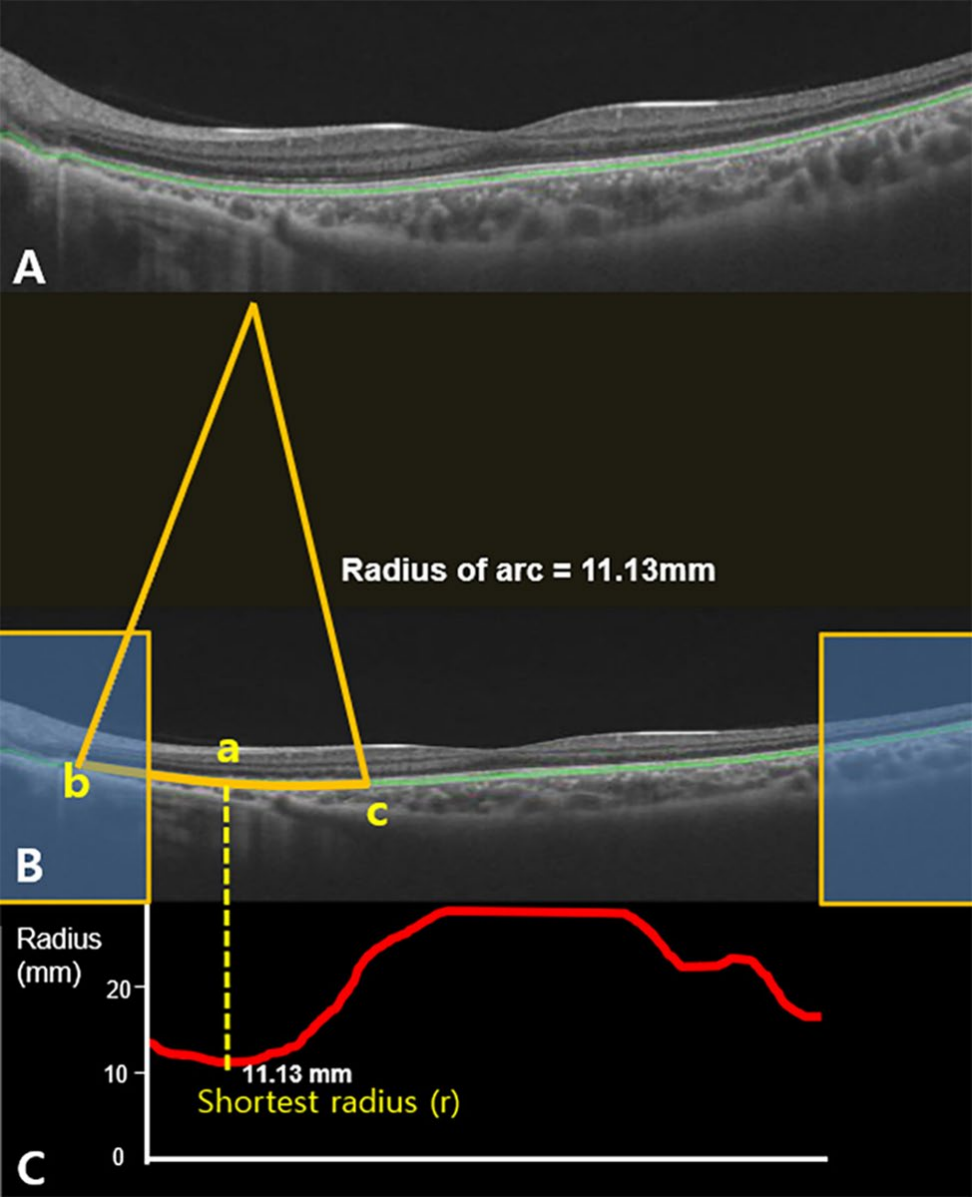
The prototype software to automatically measure radii of arcs on retinal pigment epithelium (RPE) segmentation line on OCT images. (**A**) The machine software automatically detects the RPE segmentation line (green line). (**B**) Given that the raw OCT images were elongated to twice the size in the vertical dimension in the machine software, the horizontal to vertical pixel ratio was corrected to 1:1. The region of interest was defined by excluding 100 pixels in the horizontal dimension on both sides of the image (Yellow box). The arc at the pixel (**a**) was made by connecting two pixels (**b** and **c**) apart by 1.4 mm [100 pixels apart from the pixel (**a**)] on RPE segmentation line. (**C**) The radius of arc was calculated at every pixel on the RPE segmentation line in the region of interest and they were presented as the graph (red line). Among graphs of the radii of arc (red line), the shortest radius was named ‘r’ (mm) (Fig. 1C). The average ‘r’ of the 12 radial OCT scans was designated as ‘R’ (mm)

We developed a prototype software to measure the radius of curvature on the RPE segmentation line in OCT images. Our software was developed for Windows using Microsoft Visual Studio 2015, C# language with a dot net library, and Telerik library for advanced user interface (supplementary Fig. 1). The measurements were performed on 12 9-mm radial scans. The segmentation errors were manually corrected. The software automatically detected the RPE segmentation line (Fig. 1A). As the presenting image was elongated to twice the size in the vertical dimension in the machine software, the horizontal-to-vertical pixel ratio was corrected to 1:1 (Fig. 1B). The region of interest was defined by excluding 100 pixels in the horizontal dimension on both sides of the image.

The radius of the arc was calculated at every pixel on the RPE segmentation line in the region of interest; an arc on each pixel was made by connecting two pixels that were 1.4 mm apart (100 pixels apart from the pixel) on the RPE segmentation line, and its radius was calculated by the software (Fig. 1B). Among the measured radii, the shortest radius in a single radial OCT scan image was considered as ‘r’ (mm) (Fig. 1C). The average ‘r’ of the 12 radial scans was designated as ‘R’ (mm). The reciprocal of R was multiplied by a constant (337.5). The calculated parameter was defined as the macular curvature index (MC index = 337.5/R).

The presence of posterior staphyloma was assessed by a single expert (SWP) using ultra-wide fundus photography (Optos PLC; Dunfermline, Scotland, UK). Eyes with a distinct inflection line of the surrounding macula were classified in the “definite posterior staphyloma” group. Eyes with no inflection line were included in the “no posterior staphyloma” group. If it was difficult to decide the presence of posterior staphyloma based on the ultra-wide fundus photo alone or if the presence of nasal, inferior, or peripapillary staphyloma was suspected, they were included in the “undetermined” group.

### Statistical analysis

The calculated values are presented as mean ± standard deviation. Fisher’s exact test and Mann–Whitney U test were used to compare categorical and continuous variables, respectively. The Pearson correlation coefficient test was used to determine the significance of the associations between the MC index, age, and axial length. All statistical analyses were performed using the Statistical Package for the Social Sciences for Windows 22.0 (SPSS Inc., Chicago, IL, USA). Statistical significance was defined as *P* values < 0.05.

## Results

This study involved 268 subjects. The characteristics of the study subject are listed in Table 1. The mean age of the study subjects was 60.5 ± 15.8 years, and the mean axial length was 25.416 ± 2.935 mm. The mean MC index was 33.02 ± 22.62. We were able to determine whether the presence of posterior staphyloma or not in 256 eyes (95.5%) using ultra-wide fundus photography. Among them, 54 eyes (20.1%) were included in the definite staphyloma group, and 202 eyes (75.4%) were included in the no posterior staphyloma group. In the undetermined group (*n* = 12), half of the participants were < 45 years of age. One eye was diagnosed with retinitis pigmentosa, and three eyes were suspected to have a staphyloma other than posterior staphyloma (Table 2).

**Table 1:** Summary of the age, axial length and macular curvature (MC) index in the three groups

	Number of eyes	Age	Axial length(mm)	MC index	Maximum MC index	Minimum MC index
No posterior staphyloma	202	60.9 ± 16.2	24.069 ± 1.587	20.62 ± 6.04	37.5	11.4
Definite posterior Staphyloma	54	62.2 ± 11.9	29.911 ± 2.044	71.9 ± 16.78	109.6	42.7
Undetermined	12	46.3 ± 18.2	27.863 ± 1.979	40.79 ± 9.57	55.5	21.3
Total	268	60.5 ± 15.8	25.416 ± 2.935	32.02 ± 22.62	109.6	11.4
*P* value		< 0.001	< 0.001	< 0.001		

**Table 2:** Demographic characteristics of the study subjects in the group undetermined

	Age	Axial length(mm)	MC index	Specific notification
1	8th decade	26.22	51.6	Inferior staphyloma suspected
2	4th decade	27.11	39	Young age
3	5th decade	27.22	39.6	Young Peripapillary staphyloma suspected
4	6th decade	27.29	34.5	None
5	7th decade	28.01	40.1	None
6	6th decade	28.05	21.3	None
7	5th decade	28.71	30.6	Young age
8	3rd decade	29.17	39.2	Young age
9	4th decade	30.97	49.8	Young age
10	2nd decade	31.24	55.5	Young age
11	6th decade	24.43	48.6	Retinitis pigmentosa
12	6th decade	25.93	39.7	Peripapillary staphyloma suspected

**Fig. 2:**
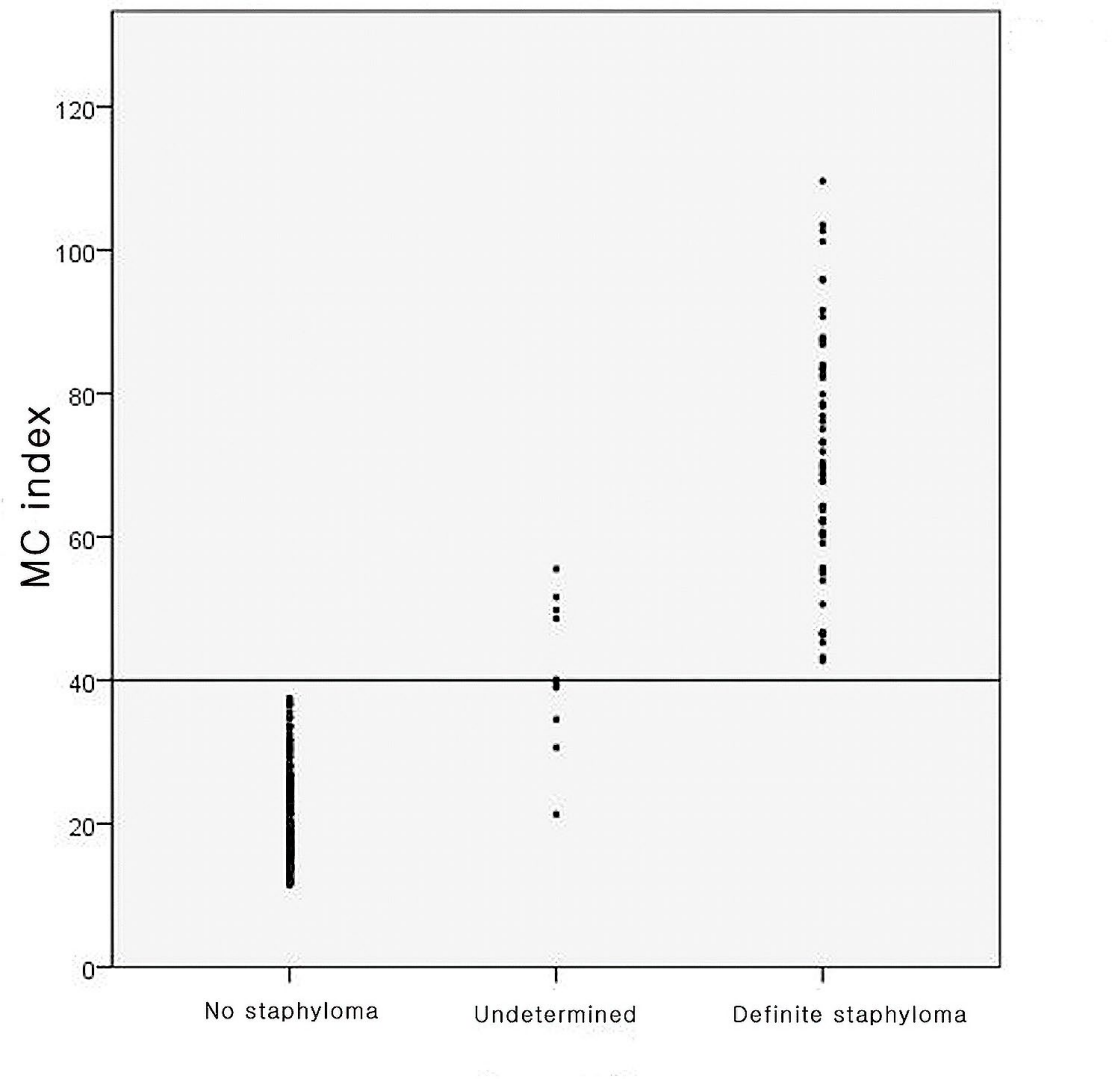
Q1The distribution of macular curvature (MC) index among the three groups. Among the 202 eyes in the no staphyloma group, the maximum MC index was 37.5. Among the 54 eyes in the definite staphyloma group, the minimum MC index was 42.7

In the no posterior staphyloma group, the average MC index was 20.62 ± 6.04, the minimum MC index was 11.4, and the maximum MC index was 37.5. In the definite posterior staphyloma group, the average MC index was 71.90 ± 16.78 and ranged from 42.7 to 109.6. In the undetermined group, the average MC index was 40.79 ± 9.57, the maximum MC index was 55.5, and the minimum was 21.3 (Table 1). These results suggest that an MC index value of 40 is suggestive of posterior staphyloma (Fig. 2).

**Fig. 3:**
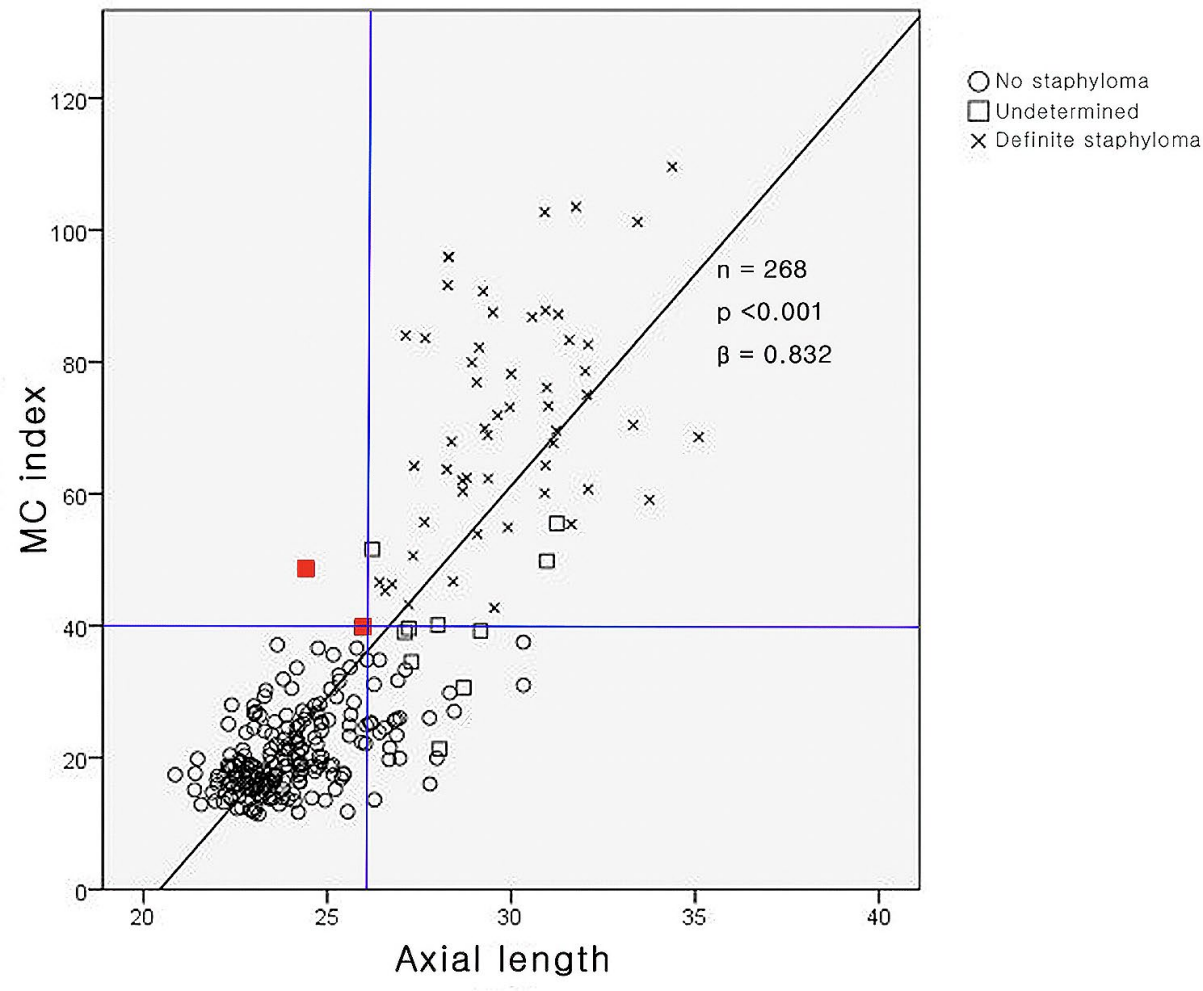
Correlation of the macular curvature (MC) index with the axial length. The MC index was observed to have a strong positive correlation with the axial length (P < 0.001, r = 0.848). All eyes (178 eyes) with axial length < 26 mm were classified in the no staphyloma group, except two eyes (red dots) in the undetermined group

The association between the MC index and axial length is shown in Fig. 3. The MC index had a strong positive correlation with axial length (*P* < 0.001, *r* = 0.848). All eyes (*n* = 178) with axial length < 26 mm were classified into the no posterior staphyloma group, except for two eyes in the undetermined group (one eye with retinitis pigmentosa with an axial length of 24.43 mm and the other with an axial length of approximately 26 mm [25.93 mm]). Eyes (*n* = 90) with an axial length > 26 mm were classified into the no posterior staphyloma (26 eyes, 28.9%), undetermined (10 eyes, 11.1%), and definite posterior staphyloma (54 eyes, 60.0%) groups.

**Fig. 4:**
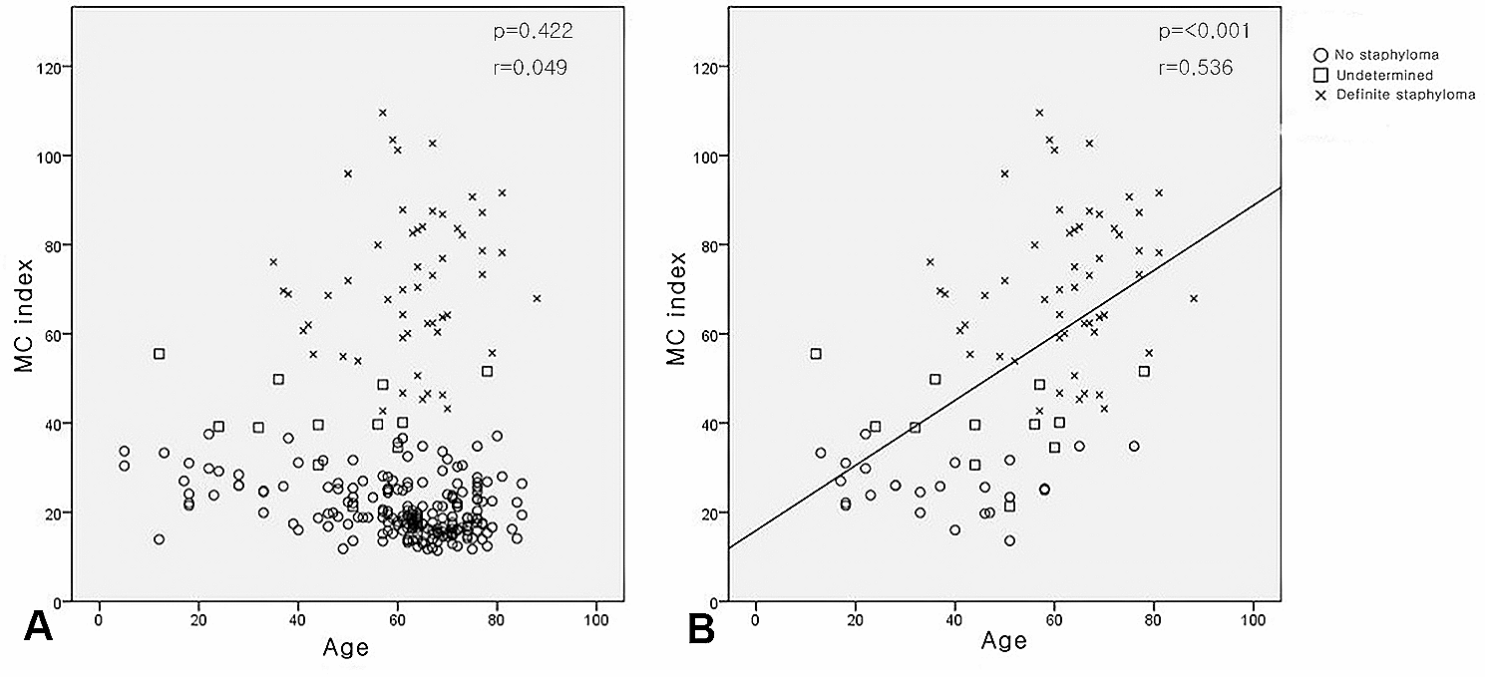
Correlation of the macular curvature (MC) index with age. (A) Correlation of the MC index with age of patients (P = 0.422, r = 0.049), and it was not significant. (B) Correlation of the MC index with age in high myopic eyes (axial length longer than 26 mm, n = 90). A strong positive correlation was observed between the MC index and age in high myopic eyes (P < 0.001, r = 0.536)

No statistically significant correlation was observed between the MC index and age (Fig. 4A, *P* = 0.422, *r* = 0.049). However, a significant correlation between the MC index and age was observed in patients with high myopia (*n* = 90; Fig. 4B, *P* < 0.001, *r* = 0.536).

## Discussion

Spaide [10] defined posterior staphyloma as, “an outpouching of the wall of the eye that has a radius of curvature that is less than the surrounding curvature of the wall of the eye”. Two main methods have been reported for diagnosing posterior staphylomas.

The first method is to identify an inflection line, which is the boundary line between the posterior staphyloma and its surrounding posterior wall. The inflection line of the posterior staphyloma can be determined using fundus photography [4, 11–14], ultrasonography [3, 15], 3D MRI [14], and ultra-widefield OCT [16–18]. Among these, ultra-widefield OCT is widely recognized as the superior method because of its ease of use and high resolution, with inflection lines displayed as outward protrusions of the sclera along with a thin choroid [16–18]. However, there are two limitations to this. First, ultra-widefield OCT is not widely available, and conventional OCT systems lack the scan length to adequately diagnose posterior staphyloma using this method. Second, as posterior staphyloma usually progresses [3], inflection lines in its early stage may not show a definite outward protrusion of the sclera or a thin choroid. Hence, ultra-widefield OCT may lack sensitivity to detect posterior staphyloma in its early stages [16]. Therefore, the lack of inflection lines on ultra-widefield OCT may not rule out the presence of posterior staphyloma.

The second method involves measuring the curvature of the posterior ocular wall. Several studies by a research group from Kyoto University [20–23] have investigated the detection of posterior staphyloma using OCT. Given the irregular curvature of the posterior wall in eyes with staphyloma, two values have been suggested to characterize the curvature of the posterior wall: the average of its total radius and the mean deviation of the posterior wall. However, this study did not report a definite reference value for the diagnosis of posterior staphyloma.

**Fig. 5:**
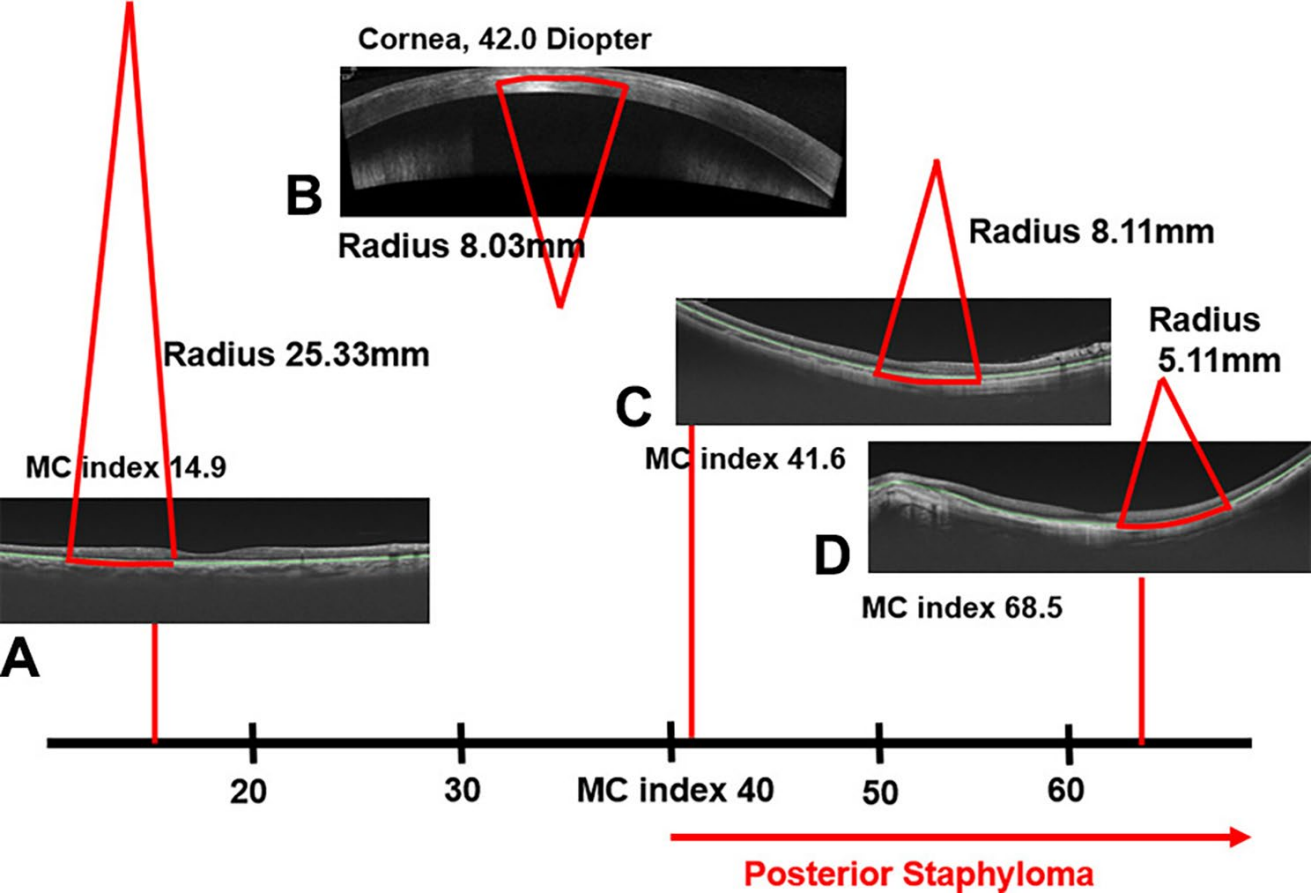
MC index: meaning and application in clinical practice. The MC index is the reciprocal of the radius of curvature at the steepest RPE segmentation line. (MC index = 337.5/R). This is similar to a formula to convert the corneal radius into corneal diopters. In the current cohort, the MC index was > 40 in all eyes with posterior staphyloma (**C, D**). This seems to be an exclusive feature compared with eyes without posterior staphyloma (**A**). A steeper retinal pigment epithelium (RPE) segmentation line indicated a higher MC index. As the principles of the two formulas are the same, it is possible to indirectly compare the curvature of the RPE segmentation line to that of the cornea measured through keratometry. The curvature of the RPE with an MC index of 41.6 (**B**) is similar to the curvature of the central cornea with 42.0 diopter (**B** and **C**)

Given that structural deformity related to posterior staphyloma should present as a steep curvature of the RPE segmentation line, the current study suggests a new marker for posterior staphyloma. It detects the steepest curvature on the RPE segmentation line and measures the radius of curvature. Focal deformities on the RPE segmentation line were neglected by the software, and the average radius (R) was obtained by averaging twelve radii from 12 9 mm-radial OCT images of each eye. The MC index was calculated by taking the reciprocal of R and multiplying by the constant of 337.5. This is similar to a formula to convert the corneal radius into corneal diopters. As the principles of the two formulas are same, it is possible to indirectly compare the curvature of the RPE segmentation line to the curvature of the central cornea by using the MC index and keratometry value (radius or diopters). For example, the steepest curvature on the RPE segmentation line with an MC index of 40 (radius, 8.44 mm) corresponds to the curvature of the central cornea with 40 diopters (radius, 8.44 mm) (Fig. 5).

The MC index was greater than 40 (radius shorter than 8.44 mm) in all eyes with definite posterior staphyloma. In contrast, eyes with no posterior staphyloma had an MC index of less than 40, suggesting that the MC index of 40 (radius, 8.44 mm) could be a potential biomarker for the diagnosis of posterior staphyloma. The MC index also has a significant correlation with axial length and age. Given the previously reported notion that the severity of posterior staphyloma is associated with age and axial length [20], the authors propose that the MC index may be utilized to monitor the severity of posterior staphyloma, although further studies are needed for confirmation.

### Limitations

This study had some limitations. To assess the suitability and efficacy of a new diagnostic tool, it must be verified using existing methods. However, data regarding ultra-wide OCTs were not available for comparison. Hence, in the present study, the MC index was verified against the findings of the ultra-wide fundus photography. The current study included a relatively small number of eyes, so not enough eyes with atypical features may have been included. In addition, it was difficult to determine the presence of posterior staphyloma in eyes with suspected peripapillary, nasal, or inferior staphylomas, or early features of posterior staphyloma. These eyes were assigned to an undetermined group and excluded from validation of the new diagnostic value. Future studies are required to elucidate the application of our method to various types of eyes, including various subtypes of staphylomas. This retrospective study was based on cross-sectional data obtained from a single referral hospital. Considering posterior staphyloma would change by aging, the findings of the current study need to be validated in future longitudinal studies.

In addition, the current cohort did not represent the characteristics of the general population, as only eyes with a documented axial length were included in the study.

## Conclusions

Eyes with posterior staphyloma have a steeper curvature than those with a radius of 8.44 mm, while eyes without posterior staphyloma do not. An MC index of 40 (radius, 8.44 mm) might act as a reference to distinguish between patients with or without posterior staphyloma. The MC index reflects the severity of posterior staphyloma; hence, it can be used as a diagnostic criterion for posterior staphyloma as well as a value to monitor its severity.

### Electronic supplementary material

Below is the link to the electronic supplementary material.

Supplementary Material 1

## Data Availability

All data generated or analyzed during this study are included in this published article.
