# Supplementary figures and images for: Diagnosis of posterior staphyloma using the radius of steepest curvature among retinal pigment epithelium segmentation line measured by optic coherent tomography
Source: BMC Ophthalmol. 2024 Feb 7;24:58. doi: 10.1186/s12886-024-03321-z (PMC10851488; doi:10.1186/s12886-024-03321-z)

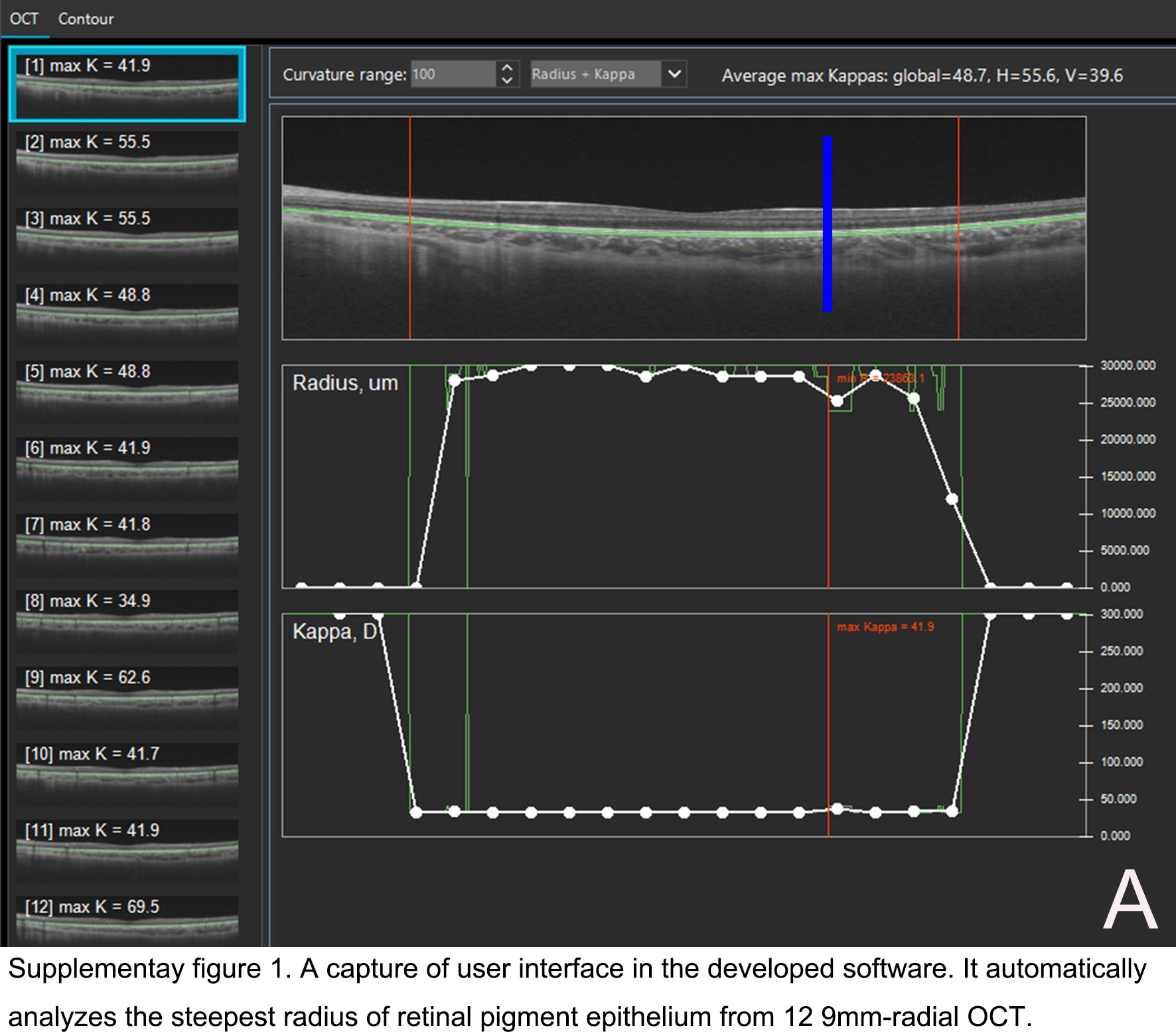

Supplement: Supplementary file 1 — Supplementary Material 1 [file 12886_2024_3321_MOESM1_ESM.jpg]
